# Biochar from Grapevine Pruning Residues as an Efficient Adsorbent of Polyphenolic Compounds

**DOI:** 10.3390/ma16134716

**Published:** 2023-06-29

**Authors:** Melissa Prelac, Igor Palčić, Danko Cvitan, Dominik Anđelini, Maja Repajić, Josip Ćurko, Tvrtko Karlo Kovačević, Smiljana Goreta Ban, Zoran Užila, Dean Ban, Nikola Major

**Affiliations:** 1 Institute of Agriculture and Turism, Karla Huguesa 8, 52440 Poreč, Croatia; melissa@iptpo.hr (M.P.); danko@iptpo.hr (D.C.); dominik@iptpo.hr (D.A.); tvrtko@iptpo.hr (T.K.K.); smilja@iptpo.hr (S.G.B.); zoran@iptpo.hr (Z.U.); dean@iptpo.hr (D.B.); nikola@iptpo.hr (N.M.); 2 Department of Food Engineering, Faculty of Food Technology and Biotechnology, University of Zagreb, Pierottijeva 6, 10000 Zagreb, Croatia; maja.repajic@pbf.unizg.hr (M.R.); josip.curko@pbf.unizg.hr (J.Ć.)

**Keywords:** adsorption, biomass valorization, phytochemicals, polarity, pyrolysis

## Abstract

Agricultural waste, which is produced in large quantities annually, can be a threat to the environment. Biochar (BC) production represents a potential solution for reducing the amount of grapevine pruning residues and, accordingly, the impact on the environment and climate change. Biochar produced by the process of pyrolysis from grapevine pruning residues was investigated and characterized to be applied as an adsorbent of polyphenolic compounds with the aim of using the waste from viticultural production to obtain a quality product with adsorption and recovery potential. Standards of caffeic acid (CA), gallic acid (GA), and oleuropein (OLP) were used as polyphenolic representatives. The obtained data were fitted with the Langmuir and Freundlich isotherms models to describe the adsorption process. The best K_L_ (0.39) and R^2^ (0.9934) were found for OLP using the Langmuir model. Furthermore, the adsorption dynamics and recovery potential of BC were investigated using an adapted BC column and performed on an HPLC instrument. The adsorption dynamics of biochar resulted in the adsorption of 5.73 mg CA g^−1^ of BC, 3.90 mg GA g^−1^ of BC, and 3.17 mg OLP g^−1^ of BC in a 24 h contact. The online solid phase extraction of the compounds performed on an HPLC instrument yielded a recovery of 41.5 ± 1.71% for CA, 61.8 ± 1.16% for GA, and 91.4 ± 2.10% for OLP. The investigated biochar has shown a higher affinity for low-polar compound adsorption and, consequently, a higher polar compound recovery suggesting its potential as an efficient polyphenolic compound adsorbent.

## 1. Introduction

According to Eurostat data [1], the European Union counted 3.2 million ha of vineyards in 2020. Annual dormant grapevine pruning is an agro-technical operation performed to maintain the shape of the vine and obtain higher quality grapes and a healthier yield [2]. In this way, the cultivation of vines itself creates huge amounts of biomass that remain after pruning. The amount of grapevine pruning residues (GPRs) produced annually ranges from 14.8 to 37 million tons globally and 38–95 thousand tons in Croatia [3]. Nowadays, pruning residue management is mostly a disposal problem [4]. The disposal of GPRs represents a cost for producers and a threat to the environment due to shredding, burring, or burning in the field, causing pathogens spreading, gas emissions in the atmosphere, and fire risk, respectively [5,6]. However, many pieces of research mentioned in a study by Jesus et al. [6] have investigated the potential of GPRs valorization in the production of oligosaccharides from hemicellulose, antioxidant compounds from lignin, organic acids, and bioethanol from saccharification of cellulose, ashes, proteins, and extractives. Furthermore, using GPRs in pyrolysis processes can reduce environmental impact, and due to chemical composition, availability, and low cost, it could have a potential application in the context of a circular economy [7].

Biochar is defined as a carbon-rich, porous material, usually obtained by the thermal decomposition of biomass under oxygen-limited conditions [8,9,10]. Different feedstocks are used for biochar production, mainly forestry waste, municipal solid waste, and agricultural residues [11,12]. Biochar is produced by the pyrolysis process wherein organic materials are heated in a partial or total absence of oxygen at different temperatures, residence times, and heating transfer rates [9,13,14]. Depending on those factors, pyrolysis can be categorized into slow and fast pyrolysis [12,15]. Slow pyrolysis or conventional carbonization is a heating process of biomass at a low heating rate for a relatively long residence time, and it yields a high amount of biochar, as previously reported [3]. On the other hand, fast pyrolysis is used to produce biochar at a high heating rate and short residence time, yielding more in bio-oil [15]. Therefore, solid, liquid, and gaseous compounds are produced during the pyrolysis process [16]. Natural polymeric compounds such as lignin and cellulose are broken in fractions during pyrolysis obtaining char, bio-oil, and incondensable gases containing carbon dioxide, hydrogen, and nitric oxide [12,17]. Biochar pyrolysis production is a simple, sustainable, and low-cost method for reducing the mass and volume of waste or residues [12,14]. The main difference between biochar and activated carbon is that biochar is obtained by biomass pyrolysis, whereas activated carbon is biochar, which has been activated chemically or physically [18]. According to several authors [8,12,15], biochar is used for soil amelioration and remediation, carbon sequestration, additive in organic solid waste composting, as decontamination of water and wastewater, activator, as electrode materials and electrode modifier, as a precursor for making catalysts and contaminant adsorbents.

Recently, biochar has been studied as an adsorbent due to its highly-porous structure, various functional groups present on the surface [9], large surface area, and easy surface modification [18]. Adsorption efficiency depends on many factors, such as pH, electrolyte content, pyrolysis temperature, surfactant structure [19], and the feedstock used in biochar production due to different chemical compositions [10,12,20]. Moreover, natural feedstock characteristics have an impact on the magnitudes of surface area, pore size distribution, cation exchange capacity, and functional groups on biochar surface, resulting in different sorption capacities [8,12]. During the carbonization process, gases are released from the material resulting in biomass porosity and total surface area changes [10]. The surface area has an important role in the determination of biochar sorption capacity [21]. Biochar surface functional groups are mostly carboxyl, hydroxyl, or formyl groups and determine the particle surface acidity and polarity (hydrophilic or hydrophobic) [10,22]. Biochars from various feedstocks have been studied as an adsorbent of pollutants, heavy metals, and organic contaminants such as pesticides, antibiotics, dyes, etc. [23]. Modified deashed wheat-straw biochar was investigated by a group of authors [24] as a pesticide adsorbent using five pesticides of various hydrophobicity and water solubility. The investigated modified biochar has shown a great potential to be applied as a superabsorbent, immobilizing organic pollutants of varied hydrophobicity from water and soil solutions. Furthermore, alkaline hydrogen peroxide pretreated sesame straw-derived biochar was used for Cd^2+^ removal from water [23]. The maximum Cd^2+^ adsorption capacity of AHP-pretreated biochar was 87.13 mg/g showing its potential as an efficient cadmium adsorbent. Vieira et al. [25] have conducted research using spent mushroom substrate biochar obtained at different temperatures to remove endocrine disruptors from water. The results showed better adsorption when biochar pyrolyzed at 600 °C was used, suggesting a good and viable option for the removal of contaminants such as hormones. As a result of its characteristics and favorable adsorption properties, biochar began to be applied in polyphenols removal [26].

Polyphenols are plant secondary metabolites with high antioxidant activity, which enables the prevention of oxidative stress related to chronic diseases [27,28]. Many papers reported their benefits on human health [29,30] and their use in the food, pharmaceutical, and nutraceutical industries [31]. Caffeic acid, or 3,4-dihydroxycinnamic acid (CA), is a hydroxycinnamic acid belonging to the phenolic acids family, ubiquitous in plant species and food, largely in coffee, wine, tea, and propolis [29]. Gallic acid or 3,4,5-trihydroxybenzoic acid (GA) is a polyphenol compound from the hydroxybenzoic acids group, mostly found in fruits, vegetables, and herbs [32,33]. According to Yang et al. [32], many studies have reported that GA has various biological properties; antioxidant, anticancer, anti-inflammatory, and antimicrobial properties. The same group of authors has stated the use of GA and its derivatives in many industries as a food supplement or additive. Oleuropein (OLP) is a secoiridoid compound produced by a plant’s secondary metabolism, and it is present in many plant species, such as *Oleaceas*, *Gentianales*, *Cornales,* and others [34,35]. OLP is an ester of 2′-(3′,4′-dihydroxyphenyl) ethanol (hydroxytyrosol) and the oleosidic skeleton common to the secoiridoid glucosides of *Oleaceae* [35]. It is mainly found in olives, olive leaves, and oil, and it has attracted attention from scientists due to its many health benefits [36]. Due to their wide distribution, easy availability in nature, application in various industries, and chemical properties, these polyphenolic compounds were used in this research.

The objectives of this work were to characterize biochar produced from grapevine pruning residues and investigate its potential in targeted polyphenol compounds adsorption using Langmuir and Freundlich isotherm models, adsorption dynamics method on HPLC (high-pressure liquid chromatography) and the recovery of valued compounds using adapted SPE method, with the aim to validate agricultural waste and obtain high-value phytochemicals.

## 2. Materials and Methods

### 2.1. Biochar Preparation

Grapevine pruning was performed in January 2021 in an experimental ‘Istrian Malvasia’ (*Vitis vinifera* L.) vineyard grafted on rootstocks 420A and SO4 at the Institute of Agriculture and Tourism in Poreč, Croatia. The GPRs were collected and left to air dry. The physicochemical characterization of GPRs was previously reported [3]. Afterward, using a Kon-Tiki system GPRs were pyrolyzed at a temperature of around 400 °C, precisely described by Cornelissen et al. [37]. The pyrolysis flame curtain consisted of adding one layer of biomass at a time. The fire started at the bottom of the cone-shaped metal system, creating the first layer, whereupon a thin layer of biomass was added to the top of the embers. When ash appeared on the outside of the carbonizing biomass, the next layer of biomass was spread homogeneously on top. Once the desired temperature and carbonizing were reached, the fire was extinguished with water. The biochar was left to cool and dry. The obtained biochar was air dried for 24 h at 105 °C (Memmert UF160, Schwabach, Germany) and afterward ground in a mortar mill (Retsch, RM 200, Haan, Germany). The obtained powder was sieved through a screen to obtain a particle size between 125 and 250 µm.

### 2.2. Biochar Characterization

Biochar yield from GRPs was calculated using the following equation (Equation (1)):

BC_yield_ = (BC_m_/GPR_m_) × 100 (1)

where BC_yield_ is the mass yield of biochar, BC_m_ is the mass of biochar in kg, and GPR_m_ is the mass of pruning residues in kg. The results are expressed in percentage (%).

The pH value was determined using a pH meter inoLab Multi 9310 IDS (Xylem Inc., Washington, WA, USA). Briefly, 5 mL of ground sample was mixed with 25 mL of 0.01 M CaCl_2_ (1:5; *v*/*v*) and rotated for 1 h. The instrument was calibrated at pH 4.01, 7.00, and 10.00 with technical buffers (WTW, Xylem Analytics GmbH, Weilheim, Germany). The analysis was conducted according to DIN ISO 10390 [38].

Electrical conductivity (EC) was measured using an EC meter (FiveGo F3, Mettler Toledo AG, Columbus, OH, USA). Briefly, 1 g of sample was mixed with 10 mL of distilled water (1:10; *m*/*v*) and rotated for 1 h to obtain a homogenized suspension.

Biochar total carbon (TC) content was determined by burning 50 mg of ground biochar on a total organic carbon analyzer (TOC-L, Shimadzu Corporation, Kyoto, Japan) connected to a solid sample combustion unit (SSM-5000A).

Biochar nitrogen content determination was performed using the Kjeldahl method [39]. First, samples (0.5 g) were digested by adding 12 mL of H_2_SO_4_ and 2 KJTabs™ tablets in each tube. Digestion was performed at 420 °C for 1 h, and samples were left to cool. Afterward, the distillation was performed on a UDK 149 Nitrogen Analyzer (VELP Scientifica Srl., Usmate Velate, Italy), with the addition of 30 mL of H_3_BO_4_ and 50 mL of NaOH. Nitrogen content was determined by titration on a SI Analytics TitroLine^®^5000 (Xylem Inc., Washington, DC, USA) using 0.1 N HCl.

Biochar samples were decomposed by microwave digestion (Ethos UP, Millestone Srl, Milan, Italy) in a two-step process. Firstly, 200 mg of samples were fused with 6 mL of HNO_3_, 2 mL of H_2_O_2_, and 0.4 mL of HF, setting microwave program at 15 min ramp to reach 190 °C and holding the obtained temperature for the next 20 min. After the first run, samples were left to cool. Finally, 5 mL of 4% H_3_BO_3_ were mixed with the first digestion samples, and microwave setting was changed in 8 min temperature ramp to 160 °C, and holding the temperature for 7 min. Biochar elemental composition was analyzed on an Inductively Coupled Plasma Emission Spectroscopy (ICP-OES) produced by Shimadzu Corporation (Kyoto, Japan) consisting of an autosampler (AS-10) and a plasma atomic emission spectrometer (ICPE-9820) using both axial and radial viewing. Samples were diluted with distilled water (1:10, *v*/*v*^−1^) before analysis.

Brunauer–Emmett–Teller (BET) method [40] was used for specific surface area (SSA) determination. Biochar samples were previously ground and dried. SSA was determined using Gemini 2380 Surface Area Analyzer (Micromeritics, Norcross, GA, USA) by nitrogen adsorption with liquid nitrogen temperature of −196 °C. A scanning electron microscope (SEM) combined with a field emission gun (QUANTA 250 FEG—SEM, FEI Company, Hillsboro, OR, USA) was used for surface morphology observation of GPRs and BC.

For investigating the functional groups present on the surface of biochar, IRTracer-100 Fourier-transform infrared spectrometer (FTIR) by Shimadzu (Kyoto, Japan) was used. Samples were ground to fine powder in a mortar and mixed with potassium bromide (1:500 mass ratio) to obtain pressed pellets. All absorptions were observed in the region from 4000 to 400 cm^−1^ using 4.0 cm^−1^ spectral resolution.

### 2.3. Adsorption Capacity

The adsorption equilibrium experiments of biochar were performed in 24 h contact with targeted standards. For this experiment, standards of CA (Sigma-Aldrich, St. Louis, MO, USA), GA (Alfa Aesar, Haverhill, MA, USA), and OLP (Sigma-Aldrich, St. Louis, MO, USA) were used. Standards solutions were prepared by dissolving 10 mg of each standard in 100 mL of distilled water to obtain a stock concentration of 100 mg L^−1^. In the first experiment, the solutions were diluted in different concentrations ranged from 5 mg L^−1^ to 50 mg L^−1^, while the biochar dosage was 1 g L^−1^, with a final reaction volume of 10 mL. In the second experiment, the solutions were diluted in concentration of 30 mg L^−1^, and biochar dosage ranging from 0.5 to 2.5 g L^−1^. The tubes were rotated for 24 h at 25 °C. Aliquot reaction solutions were collected and quickly filtered through a 0.22 μm filter. The analyzes were performed on a Shimadzu Nexera UPLC-PDA instrument consisting of a degassing unit (DGU-405, Shimadzu, Kyoto, Japan), an autosampler (SIL-40CX3, Shimadzu, Kyoto, Japan), a system controller (SCL-40, Shimadzu, Kyoto, Japan), a photodiode array detector (SPD-M40, Shimadzu, Kyoto, Japan), two solvent delivery units (LC -40DX3, Shimadzu, Kyoto, Japan), a column oven (CTO-40C, Shimadzu, Kyoto, Japan), and a Poroshell 120 EC-C18 2.7 µm column (2.1 mm × 150 mm) (Agilent, Palo Alto, CA, USA). The temperature in the column oven was held at 40 °C. The injection volume was 5 µL, with a flow rate set at 0.4 mL min^−1^. Gradient elution was performed as follows: 0–4 min 95% A to 5% B, 4–4.10 min 5% A to 95% B, and 4.10–7 min 95% A to 5% B, where solvent A was water and solvent B was methanol, both containing 0.1% acetic acid. The total run time was set to 7 min. Calibration curves were obtained by injecting serial standards dilutions of CA (y = 7505.21x + 0, R^2^ = 0.9999), GA (y = 6300.54 × −8335.94, R^2^ = 0.9997), and OLP (y = 566.84 × −676.33, R^2^ = 0.9998). Identification and quantification of standards were performed at 280 nm.

The results of the first experiment were fitted in the Langmuir and Freundlich isotherms. The Langmuir isotherm is described as the following equation (Equation (2)) [41,42]:

1/q_eL_ = 1/q_max_ + 1/(K_L_ × q_max_) × 1/γ_e_ (2)

where q_eL_ is the amount of adsorbate concentration in the solid phase at equilibrium (mg g^−1^), 1/q_max_ is the slope of linear equation, 1/(K_L_ × q_max_) is the y-intercept, K_L_ is the affinity constant (L mg^−1^), q_max_ is the maximum monolayer adsorption capacity (mg g^−1^), and γ_e_ is the amount of adsorbate concentration in the liquid phase at equilibrium (mg L^−1^). The equation was plotted as 1/q_eL_ vs. 1/γ_e_, and obtained R^2^ was used as coefficient of determination, which is an indicator of representativeness of the model. The closer the values are to 1, the more representative the model is. Furthermore, the R_L_ factor was calculated to determine the auspicious and inauspicious of Langmuir isotherms as described in Equation (3) as follows:

R_L_ = 1/(1 + K_L_ × γ_0_) (3)

where K_L_ is the affinity constant (L mg^−1^), and γ_0_ is the initial concentration of the adsorbate (mg L^−1^). The result 0 < R_L_ < 1 indicates a favorable adsorption, R_L_ > 1 unfavorable, R_L_ = 1 a linear, and R_L_ = 0 an irreversible adsorption.

The Freundlich isotherms were calculated as described in Equation (4) and plotted as log q_eF_ vs. log γ_e_. The Freundlich isotherm constant (K_F_/(mg g^−1^) × (L g^−1^)^n^), adsorption intensity (n), and R^2^ were obtained using the plot.

log q_eF_ = log K_F_ + 1/n × log γ_e_ (4)

A 4.6 mm × 50 mm stainless steel column was used to investigate the adsorption dynamics of biochar. In repeated experiments, the column was filled with 0.263–0.306 g of biochar and connected to an HPLC-UV-Vis instrument consisting of a solvent delivery module (Model 220/230/240, Varian ProStar, Palo Alto, CA, USA), UV-Vis detector (325 LC detector, Varian ProStar, Palo Alto, CA, USA), and an autosampler (Model 410, Varian ProStar, Palo Alto, CA, USA). Prior to the adsorption experiment, the biochar column was rinsed with 50 mL of HPLC-grade water, followed by 50 mL of methanol and 50 mL of acetonitrile, and preequilibrated with HPLC-grade water. The flow rate was set to 0.5 mL min^−1^. Standards solutions of CA, GA, and OLP were prepared by dissolving 2.5 mg of each standard in 250 mL of distilled water to obtain a final concentration of 10 mg L^−1^. The inlet of the solvent delivery module and the outlet of the biochar-packed column were immersed in the standard solution. The adsorption experiment was performed by continuously loading the biochar in the column with each of the dissolved standards using the solvent delivery module over the period of 24 h with the flow set to 0.5 mL min^−1^. Sampling was performed as follows: 100 µL of the sample was taken before experiment, then after 10 min, and 1, 2, 3, 5, and 24 h. The solution was stirred continuously during the run using a magnetic stirrer. The procedure was performed for all investigated standard solutions individually.

Collected samples were analyzed as described in the adsorption experiment (Section 2.3). Identification and quantification of standards were performed at 280 nm. The amount of each standard adsorbed per g of biochar in 24 h was calculated using the following formula (Equation (5)):

Q = (m_0_ − m_eq_)/m (5)

where Q is the adsorption capacity expressed as mg of standard per g of biochar, m_0_ is the mass of standard in initial elution (mg), m_eq_ is the mass of standard measured after 24 h (mg), and m is the mass of biochar in the column (g).

### 2.4. Recovery

An adapted online SPE (solid phase extraction) method was used with the aim to determine the recovery of the investigated standards on a UPLC-DAD instrument consisting of a degassing unit (DGU-405, Shimadzu, Kyoto, Japan), an autosampler (SIL-40CX3, Shimadzu, Kyoto, Japan), a system controller (SCL-40, Shimadzu, Kyoto, Japan), a photodiode array detector (SPD-M40, Shimadzu, Kyoto, Japan), two solvent delivery units (LC-40DX3, Shimadzu, Kyoto, Japan), and a column oven (CTO-40C, Shimadzu, Kyoto, Japan). The experiment was carried out by loading the biochar column with 10 µL of standard solution (10 mg L^−1^) with the flow rate set at 0.21 mL min^−1^. After the loading, the column was washed with 3 column volumes of HPLC-grade water. Subsequently, the loaded standard was eluted with 3 column volumes of methanol, and the elute was collected in an HPLC vial. The elution of the standard was monitored at 280 nm. The quantification of the eluted standard was performed as described in the adsorption experiment (Section 2.3). Separate recovery experiments were carried out for each standard. The recovery of each standard after the adapted online SPE method was calculated using the following formula (Equation (6)):

Recovery = (m_e_/m_c_) × 100 (6)

where m_e_ is the mass of the standard after elution (mg), and m_c_ is the mass of the standard load on the column of standard in initial elution (mg). The results are expressed in percentage (%).

### 2.5. Statistical Analysis

The experiment was performed in three repetitions for each standard. Descriptive statistics of the data were obtained by Statistica 13.4 (Tibco, Inc., Palo Alto, CA, USA).

## 3. Results

### 3.1. Physicochemical Characterization of GPRs Biochar

**Figure 1:**
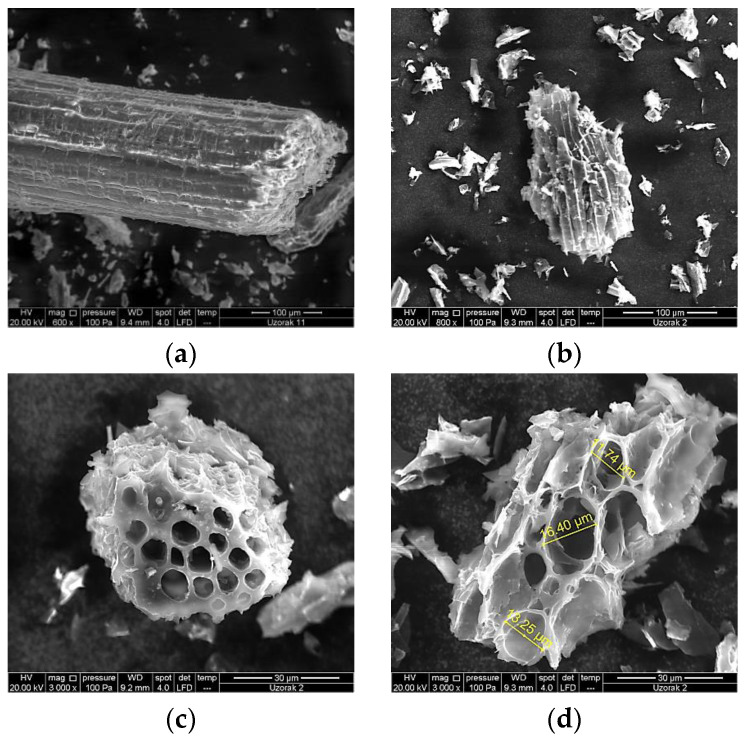
Scanning electron microscope (SEM) pictures of GPRs and biochar obtained from GPRs: (**a**) SEM magnification 600×, visible GPRs particles size of 100 µm; (**b**) SEM magnification at 800×, visible BC particles size of 100 µm; (**c**) SEM magnification at 3000×, visible BC particles size of 30 µm; (**d**) SEM magnification at 3000×, visible BC pores size.

A summary of physicochemical analyses performed on produced biochar is given in Table 1. Biochar obtained by GPRs pyrolysis showed an alkaline pH reaction and high carbon content. The most abundant macroelement in biochar was potassium, and silicon was a microelement representative. The lowest elements detected were sodium and molybdenum, respectively. The SEM measured a pores diameter ranging from 11.74 to 16.40 µm, as shown in Figure 1.

**Figure 2:**
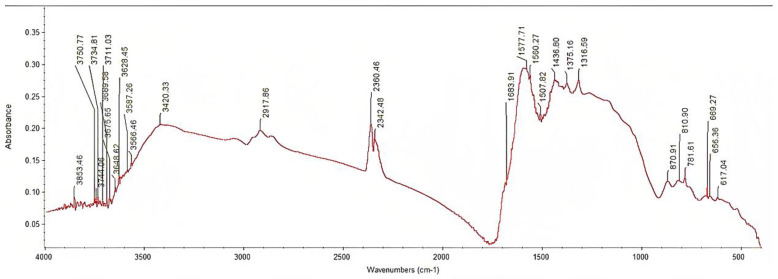
Fourier transform infrared spectra (FTIR) of GPRs biochar obtained by pyrolysis.

FTIR spectra obtained for the investigated biochar are presented in Figure 2. Table 2 describes band assignments observed on the biochar surface based on previous studies. High-intensity peaks were observed from 3420.33 to 1316.59 cm^−1^, with a decreasing rate from 3853.46 to 3566.46 cm^−1^. Bands from 3853.46 to 3566.46 cm^−1^ are connected to hydroxide bonds, while the peak at 3420.33 cm^−1^ is identified as H-bonded O-H stretching vibrations of hydroxyl groups from alcohols, phenols, and organic acids. A C-H stretching of alkyl structures and –CH_2_– asymmetric stretch is detected at 2917.8 cm^−1^. Bands from 2360.46 to 2342.48 cm^−1^ are identified as CO_2_ adsorption and carbonyl bond group. Aromatic and olefinic C=C vibrations, C=O amide(I), ketone and quinone groups, C=O vibration in a carboxylic group of phenolic acids (p-hydroxybenzoic acid), and C=C and C=O stretching are identified at 1683.91 cm^−1^. Bands from 1577.71 to 1436.80 cm^−1^ are all attributed to C=C stretching. Additionally, the peak at 1577.71 cm^−1^ is also connected to COO- asymmetric stretching, C=O, and aromatic skeletal vibration with C=O stretching vibration; 1560.27 cm^−1^ is also identified as COO- asymmetric stretching and vibrations; 1507.82 cm^−1^ is also connected to secondary aromatic amines, and 1436.80 cm^−1^ is also being identified as C-H deformation, asymmetric in –CH_3_ and –CH_2_– (cellulose). A CH deformation (cellulose and hemicellulose) is detected at 1375.16 cm^−1^, while C-O and phenyl group-CHR-OH deformation at 1316.59 cm^−1^. Bands from 870.91 to 781.61 cm^−1^ are all connected to =C-H bending. Additionally, the peak at 870.91 cm^−1^ is also connected to symmetric C-O stretching and aromatic C-H groups, C-O-C stretch, and C-H out-of-plane bending, peak at 810.90 cm^−1^ is also identified as C-O-C aromatic ethers, symmetric stretch, while the peak at 781.61 cm^−1^ is also associated to C-C alkanes skeletal vibrations, C-H and O-H out of plane bending, and Si-O stretching bands. Peaks from 656.36 to 617.04 cm^−1^ are connected to Si-O stretching bands as well.

### 3.2. Adsorption Isotherms

**Figure 3:**
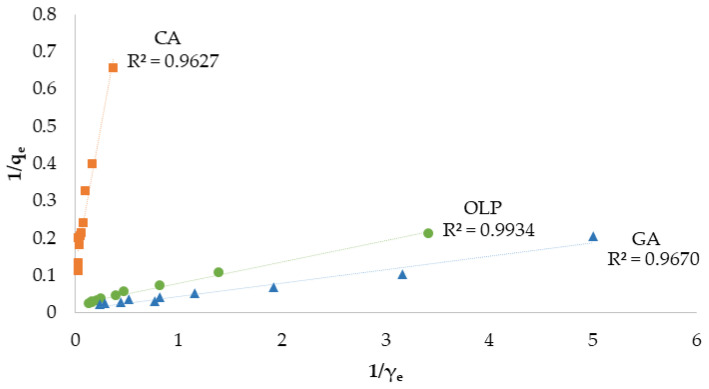
Langmuir isotherms of adsorption of CA, GA, and OLP by GPRs biochar (CA—caffeic acid, GA—gallic acid, OLP—oleuropein, R^2^—coefficient of determination, q_e_—amount of adsorbate concentration in the solid phase at equilibrium (mg g^−1^), γ_e_—amount of adsorbate concentration in the liquid phase at equilibrium (mg L^−1^)).

**Figure 4:**
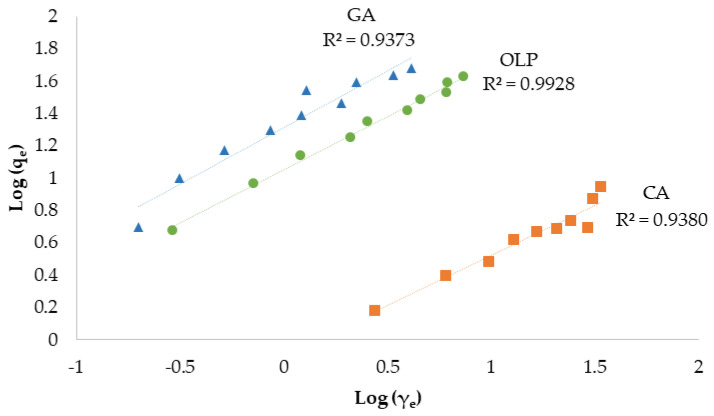
Freundlich isotherms of adsorption of CA, GA, and OLP by GPRs biochar (CA—caffeic acid, GA—gallic acid, OLP—oleuropein, R^2^—coefficient of determination, q_e_—amount of adsorbate concentration in the solid phase at equilibrium (mg g^−1^), γ_e_—amount of adsorbate concentration in the liquid phase at equilibrium (mg L^−1^)).

The results of the first experiment were fitted in the Langmuir and Freundlich isotherm models. In the Langmuir models, obtained determination coefficient values (R^2^) ranged from 0.9627 to 0.9934 (Figure 3), while in Freundlich’s ranged from 0.9373 to 0.9928 (Figure 4).

Still, Langmuir’s model showed a better fit compared to Freundlich’s model in regard to all investigated standards, with the OLP coefficient of determination reaching the highest value (0.9934). Comparing the q_max_ value from the Langmuir isotherm, GA had the highest maximum monolayer adsorption capacity (118 mg g^−1^), followed by OLP (45.5 mg g^−1^) and finally CA (8.21 mg g^−1^), as shown in Table 3. The sorption capacity calculated by K_L_ value was higher in the case of OLP, followed by GA and CA. The R_L_ coefficient was calculated using initial concentration γ_0_ (between 5 mg L^−1^ and 50 mg L^−1^), and it was in a range from 0.02 to 0.71 for the investigated standards. The Freundlich isotherm model has shown a higher adsorption capacity for GA and the lowest for CA. The value of 1/n was similar for all investigated compounds and ranged between 0.62 and 0.70. OLP showed the highest determination coefficient (0.9928) among the investigated compounds in the Freundlich model as well.

**Figure 5:**
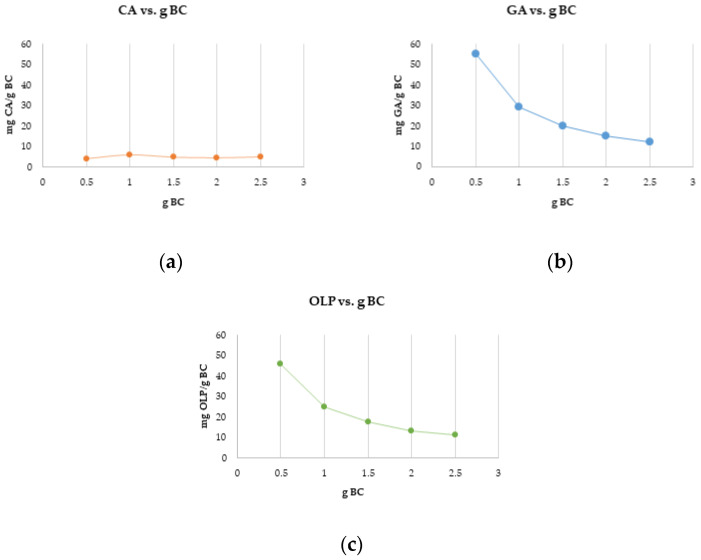
The influence of different amounts of biochar (BC) on different polyphenol adsorption: (**a**) different biochar dosages using 30 mg of caffeic acid L^−1^; (**b**) different biochar dosages using 30 mg of gallic acid L^−1^; (**c**) different biochar dosages using 30 mg of oleuropein L^−1^.

The results of the experiment with different biochar dosages using the same concentration of standards are shown in Figure 5 and expressed as mg of CA, GA, and OLP adsorbed per gram of biochar. The amount of biochar did not affect the adsorption of CA. However, the dose of 0.5 g of biochar adsorbed the highest concentrations of GA and OLP, with a decreasing trend of adsorption as the amount of biochar increased.

### 3.3. Adsorption Dynamics and Solid Phase Extraction Capacity

**Figure 6:**
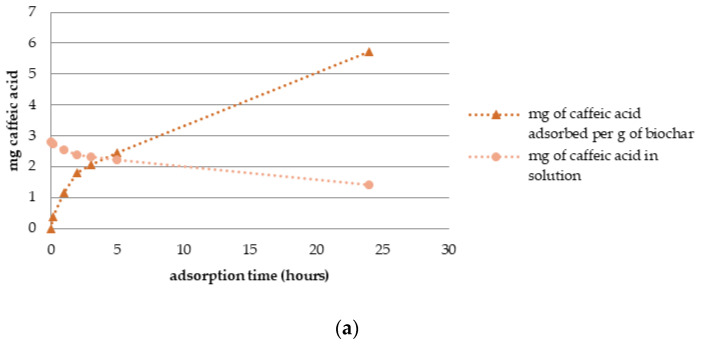

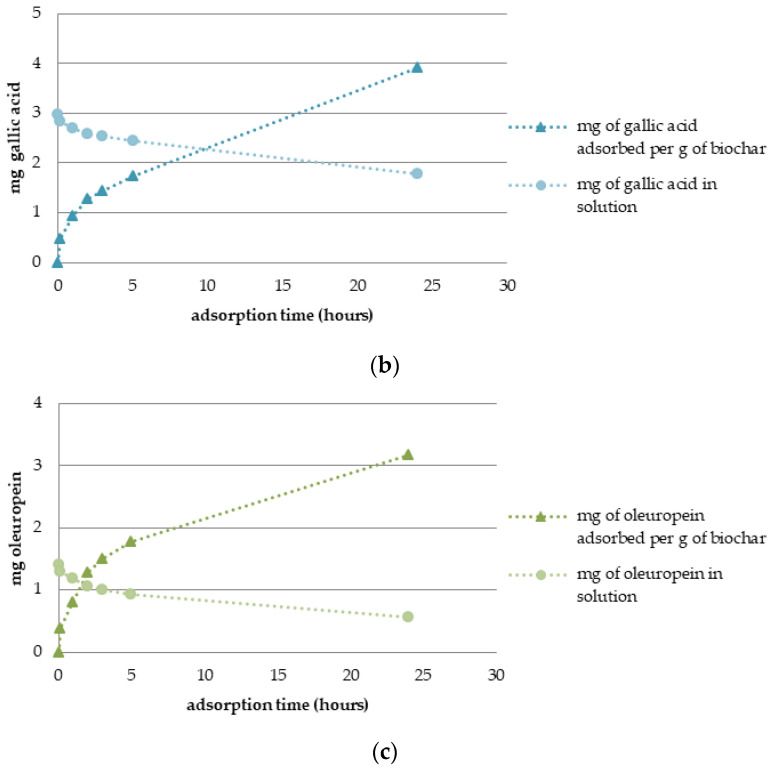
Adsorption dynamics of polyphenolic compounds on GPRs biochar (BC) (**a**) caffeic acid adsorption on BC in 24 h; (**b**) gallic acid adsorption on BC in 24 h; (**c**) oleuropein adsorption on BC in 24 h.

The adsorption dynamics results are expressed in mg of adsorbed standard over a period of 24 h. Figure 6 shows a decreasing concentration of each standard in the solution and the proportional absorbed compound quantity on the biochar column, respectively. The column in the experiment with CA was filled with 0.244 g of biochar. The initial injected solution contained 2.83 mg of CA. In 24 h, the amount of standard measured in the solution was 1.43 mg, resulting in 1.40 mg of adsorbed CA on GPRs biochar in the column. Subsequently, it was calculated that 1 g of biochar had adsorbed 5.73 mg of CA in 24 h contact.

Furthermore, the column in the experiment with GA was filled with 0.306 g of biochar. The initial injected solution contained 2.99 mg of GA. In 24 h, the amount of GA in solution exhibited a downward trend reaching 1.79 mg and resulting in 1.30 mg of the adsorbed standard on GPRs biochar in the column. Finally, the calculation of absorbed GA on biochar showed adsorption of 3.90 mg of GA per g of biochar in 24 h contact. Lastly, the column in the experiment with OLP was filled with 0.263 g of biochar. The initial injected solution contained 1.40 mg of OLP. In 24 h, the amount of OLP measured in the solution was 0.57 mg, resulting in 0.83 mg of the adsorbed standard on GPRs biochar in the column. Subsequently, it was calculated that 3.17 mg of OLP was absorbed per g of biochar in 24 h contact.

**Figure 7:**
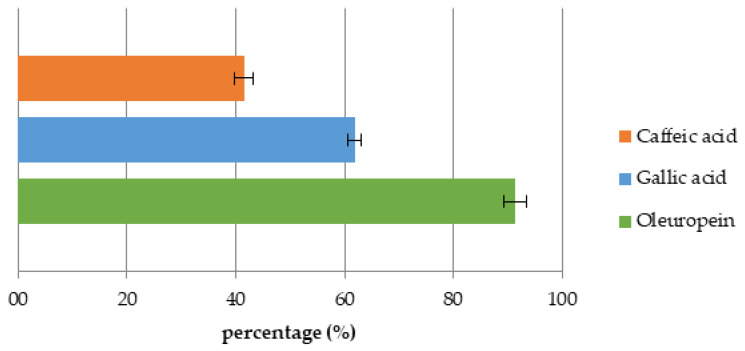
Standards recovery from GPRs biochar column.

The online solid phase extraction capacity of the GPR biochar was tested by calculating the recovery values after column loading with each investigated compound. The recovery values of the investigated standards were 41.5 ± 1.71% for CA, 61.8 ± 1.16% for GA, and 91.4 ± 2.10% for OLP (Figure 7).

## 4. Discussion

In this research, GPRs were used to produce biochar by the process of pyrolysis in a Kon-Tiki system. The pyrolysis temperature was around 400 °C. The processes of dehydration, decarboxylation, and decarbonylation of biomass occur at temperatures below 600 °C [43]. According to Tan et al. [44], biochar produced at 400 °C has reached the optimal quality for application into the soil when compared to higher temperatures. The value of pH, along with EC, are important parameters when using biochar as a soil amendment, but also for understanding the influence of these factors on phytochemical adsorption. Mostly, biochar produced from GPRs at 400 °C has an alkaline pH value [45], which was confirmed in this study. Alkaline pH can be related to high pyrolysis temperature and ash content [46], leading to an increase in base cations and carbonates [47] and loss of acidic functional groups [9]. Higher levels of biochar EC can be associated with its content of alkali and alkaline-earth elements [48,49]. In this work, K was the most abundant element in biochar, followed by Ca, P, Mg, and Si. Nitrogen content was low, while carbon content was high, as expected. Similar results were obtained by Marshall et al. [45], where biochar produced from grapevine cane at 400 °C showed values of 1.21% N and 70.5% °C. The concentration of mentioned macro- and microelements increased due to the carbonization process [50], as reported in our previous work [3]. Furthermore, the most visible change in biomass properties during pyrolysis is carbonization, leading to carbon content increase due to functional group separation. Pyrolysis temperature and carbon content are positively correlated [3]. Therefore, as the temperature of the reaction increases, so does the carbon content by decreasing oxygen and hydrogen [10,45]. According to Tomczyk et al. [20], biochar produced from woody biomass contains more carbon when compared to other materials, such as manure. The elemental composition of biochar can affect the adsorption of various compounds through both co-precipitation and inner sphere complexation [51].

Pruning residues are lignocellulosic materials [52] rich in lignin, hemicellulose, and cellulose [44]. During pyrolysis, the feedstock properties change due to the decomposition of hemicellulose and partial decomposition of cellulose and lignin, and it usually occurs at temperatures from 200 to 400 °C [53]. Pores dimensions are affected by the feedstock composition of lignin and cellulose. In fact, raw material with greater lignin content would yield biochar with a macroporous structure, whereas higher cellulose content would give a microporous structure [54]. Pores can be divided into several groups depending on their diameter size; nanopores <0.9 nm, micropores <2.0 nm, mesopores 2–50 nm, and macropores >50.0 nm providing a huge internal surface area that affects the ability of adsorption [9,19]. Furthermore, measured pore groups of biochar are divided into three size ranges considering function; transmission pores (≥50 μm), storage pores (0.5–50 μm), and residual pores (<0.5 μm) [55]. Wildman and Derbyshire [56] indicated that macroporosity is primarily caused by the pit fields and cell cavities of the feedstock. Indeed, according to the SEM results, GPRs biochar is composed of macropores (>50.0 nm) with a storage function (0.5–50 µm), suggesting that it could be used as an efficient adsorbent and nutrient recovery material. However, due to various pore sizes ranging from nanometers to micrometers, an accurate characterization of biochar pores is challenging [55]. The influence of pore size on organic compound adsorption was reported by many authors [57,58]. It is described as a pore-filling mechanism with a molecular sieving effect; organic molecules are largely restricted to access into the pores those diameters since they are smaller than the molecule diameters. Therefore, the adsorbate properties should be considered to accomplish an efficient adsorption and adapt the feedstock and production process accordingly. In this work, biochar SSA measured by BET was very low when compared with other biochar; tea waste 342.22 m^2^ g^−1^ [59], lodgepole pine wood 152 m^2^ g^−1^ [60], or activated carbon with a range from 800 to 1200 m^2^ g^−1^ [19]. However, low results were also obtained by de la Rosa et al. [61] for grapevine wood biochar, reporting an SSA value ≤5 m^2^ g^−1^ and relating it to the production conditions of biochar.

Biochar adsorptive capacity can be affected by the types of functional groups on its surface [12,62,63], referable to biochar volatile matter and oxygen contents [64]. The polarity and hydrophobicity of functional groups on the biochar surface affect the adsorption capacity of aromatics via H-bonding [63]. The larger molecular polarity of the functional groups of biochar suggested a greater adsorption potential [65]. The hydrophobic sorption on the biochar surface presumably affects the adsorption of phenols on biochar due to carbon increase content and the number of oxygenated functional groups [66,67,68]. According to Guo et al. [69], biochar has adsorbed non-polar organic compounds through pore filling, partition, and hydrophobic effect, and polar organic compounds through H-bonding, electrostatic attraction, specific interaction, and surface precipitation from the soil. Identified carbon atoms present on the studied biochar surface have the possibility to transfer electrons with targeted molecules to form active sites, which is conducive to chemical adsorption [70]. Although the hydroxy groups showed the lowest peaks in the results, they play an important role in increasing the adsorption energy of phenols on the biochar surface [22]. The surface functional groups containing oxygen affect the adsorptive properties of activated carbons [71]. The highest peaks observed from FTIR spectra are potentially identified as COO-, C=O, and C=C stretching and vibrations. As Zhou et al. [65] have reported, C=O and single-bond oxygen-containing groups are polar molecules, and their adsorption potential affected by polarity is stronger than molecules with aromatic nuclei. The COO- functional groups lead to a pH increase [72], which agrees with the alkaline result in this work. Peaks at 1436.80 and 1375.16 cm^−1^ were identified as C-H deformation, asymmetric in –CH_3_ and –CH_2_– (cellulose), and CH deformation (cellulose and hemicellulose), indicating the presence of woody and cellulosic components due to incomplete degradation of the canes [73]. Bands from 781.61 to 617.04 cm^−1^ indicate the presence of silicon monoxide attributable to biochar ash content [74]. However, the mechanisms of functional groups present on biochar surfaces in polyphenolic adsorption are uncertain due to the complexity and hard determination of the groups. Nevertheless, biochar with a high surface area and abundant functional groups is considered suitable for adsorption implementation [50].

Biochar is a relatively novel adsorbent material mainly used in pollutants, heavy metals, and emerging contaminants removal from the environment [75,76]. Huggins et al. [60] investigated the adsorption capacity between granular wood-derived biochar and granular activated carbon for its implementation in packed bed column filters for enhanced wastewater treatment and nutrient recovery. Biochar obtained a greater adsorption capacity at higher concentrations of the applied chemicals when compared to activated carbon, suggesting its potential as an adsorbent. As described, many biochar characteristics and parameters affect its adsorption potential. In this work, biochar was used for the targeted compounds’ adsorption and their recovery. Standards of CA, GA, and OLP were used as adsorbates due to their wide presence, health benefits, and potential widespread use. Biochar is a hydrophobic, non-polar material with a great affinity to low-polar molecules and favorable characteristics for application in phytochemicals adsorption. To determine and better understand the adsorption capacity of biochar, the obtained data were fitted with the Langmuir and Freundlich isotherm models. Both models were suitable for the experiment according to obtained high coefficients of determination for each compound. Still, in both models, OLP had reached a higher R^2^ value compared to both CA and GA. According to the obtained results in Langmuir’s model, the values for maximum monolayer adsorption capacity have shown that the adsorption of GA was more favorable compared to the other investigated compounds, suggesting a main monolayer adsorption reaction including the physicochemical adsorption equilibrium mechanism [77]. However, the sorption capacity calculated as K_L_ of OLP was within the values reported in Lawal et al. [78], while the difference in values obtained for CA and GA may be due to the specificity of the compounds. The R_L_ coefficient was 0 < R_L_ < 1 for all investigated standards indicating favorable adsorption. The Freundlich constant showed the affinity of GPR biochar for GA, which was 25-fold higher compared to CA. The 1/n values of all three standards were comparable and in a range from 0 to 1, indicating a similar adsorption intensity, mostly due to biochar properties [78] and suggesting chemical adsorption on the biochar surface [77]. The effect of different biochar dosages on CA, GA, and OLP adsorption was also studied. The results for CA have shown no influence of BC dosage on adsorption. Meanwhile, GA and OLP have shown similar results. The highest amount of GA and OLP per gram biochar was adsorbed at the lowest applied dose (0.5 g biochar L^−1^) and decreased with the increasing adsorbent dose, perhaps due to the saturation of the adsorbate. Similar results were obtained in the experiment conducted by Lee et al. [26], in which the lower dosage of food waste-based biochar adsorbed the highest amount of phenol. Hamzah et al. [79] explained that increasing the dosage after reaching the adsorptive optimum level could lead to adsorbent accumulation itself and overlaying the active site resulting in decreased removal efficiency. As for the adsorption dynamics experiment and the online solid phase extraction, OLP was the poorest adsorbed compound, albeit with the highest recovery, followed by GA and CA. Those results suggest the connection between the hydrophobic nature of biochar and the compound’s solubility in water. Correspondingly, OLP is a polar phenolic compound [80], while CA is a low-polar compound [81]. CA has a lower solubility in water, making it harder to extract from biochar. In a research conducted by Richard et al. [82], the adsorption potential of commercial active charcoal for phenolic compounds was investigated using CA, among others. The adsorption capacity of CA on Active Charcoal TE80^®^ (AC) was 229 mg kg^−1^ AC, which is almost 40-fold higher in comparison with the results obtained in this work. Furthermore, in another research [79], coconut shell-based activated carbon was used to determine its adsorption potential in phenolic compound removal using GA as a phenolic representative. The authors observed a few parameters that influenced the removal of GA; adsorbent dosage, initial concentration, contact time, and pH, concluding that the acid-treated activated carbon had successfully removed 97% of GA under specific conditions. Furthermore, Ekinci [83] has investigated the removal potential of commercial activated charcoal on phenolic compounds from apple juice. GA and CA were investigated, among other compounds. The loss (%) in GA and CA contents in juice samples treated with 3.0 g L^−1^ activated charcoal were found to be 41.04 and 44.26%, respectively. The value of biochar from different biomass was confirmed in Abid et al. [84] study, using biochar from olive mill solid waste for polyphenols recovery or removal from olive mill wastewater. The maximum polyphenols adsorption at 30 °C was 140.47 mg g^−1^. Finally, the experimental data indicate that GPR biochar has great potential in the adsorption of phenolic compounds as well as its application as an adsorbent in online solid phase extraction of highly valuable phytochemicals suggesting its application in wastewater or olive mill wastewater treatments [85,86].

## 5. Conclusions

Biochar from grapevine pruning residues poses a potential solution for agro-waste valorization in a number of ways; it is produced using pruning residues, and if improperly disposed of, it can cause environmental damage. Due to its characteristics favorable for adsorption, it is a potential material in high-value phytochemicals removal and recovery from agricultural and food waste. In this research, the adsorption and recovery of targeted phenolic compounds were performed using agricultural waste, sustainable and environmentally friendly procedures aligning with the green economy principles. Biochar obtained from grapevine pruning residues was investigated and characterized to determine its potential in the adsorption and recovery of caffeic acid, gallic acid, and oleuropein. The feedstock choice and production conditions are crucial in achieving desirable biochar characteristics for adsorption. Physicochemical characteristics such as pH, electrical conductivity, macro- and micronutrients, nitrogen, and carbon content, as well as its porous structure, specific surface area, and functional groups, play a main role in adsorption capacity determination. The studied biochar has adsorbed the greatest amount of caffeic acid in the adsorption dynamics experiments (5.73 mg CA g^−1^ of BC), followed by gallic acid and oleuropein. Moreover, oleuropein had reached the highest R^2^ (0.9934) and K_L_ (0.39) value in the Langmuir isotherm model and the greatest percentage of recovery (91.4 ± 2.10%). Biochar from grapevine pruning residues has shown an affinity in low-polar compounds adsorption and, consequently, a higher polar compounds recovery. Due to its properties, it could be applied in environmental remediation such as wastewater or contaminated soil treatments and phytochemicals recovery in order to obtain clean and highly valued compounds, which can be used in various industries. However, further research is needed to determine its potential in the adsorption and recovery of phytochemicals from agro and food wastes.

## Figures and Tables

**Table 1:** Physicochemical characteristics of the GPRs biochar.

Parameter	Value	Unit
yield	30.8 ± 0.02	%
pH	8.67 ± 0.04	-
EC	2.63 ± 0.29	mS cm^−1^
TC	77.15 ± 0.52	%
N	1.11 ± 0.00	%
P	2.90 ± 0.13	g kg^−1^
K	27.1 ± 1.26	g kg^−1^
Ca	17.8 ± 0.56	g kg^−1^
S	0.76 ± 0.02	g kg^−1^
Mg	2.89 ± 0.09	g kg^−1^
Na	0.03 ± 0.01	g kg^−1^
Fe	230 ± 8.70	mg kg^−1^
Al	295 ± 15.5	mg kg^−1^
Cu	19.9 ± 0.95	mg kg^−1^
Mn	58.8 ± 2.01	mg kg^−1^
Mo	2.15 ± 0.73	mg kg^−1^
Si	1706 ± 25.8	mg kg^−1^
Zn	62.7 ± 1.75	mg kg^−1^
Surface area (BET)	1.89 ± 0.01	m^2^ g^−1^

Results are expressed as mean ± standard deviation.

**Table 2:** FTIR wave number identification of GPRs biochar.

Wave Number (cm^−1^)	Range (cm^−1^)	Corresponding Vibration
617.04–656.36	460–800	Si-O stretching bands
781.61	460–800	Si-O stretching bands
	720–750	C-C alkanes skeletal vibrations
	675–1000	=C-H bending
	750	C-H out of plane bending
	650–770	O-H out of plane bending
810.90	810–850	C-O-C aromatic ethers, symmetric stretch
	675–1000	=C-H bending
870.91	885	symmetric C-O stretching and aromatic C-H groups
	875	C-O-C stretch
	675–1000	=C-H bending
	830–874	C-H out of plane bending
1316.59	1260–1350	Phenyl group-CHR-OH deformation
	1000–1300	C-O
1375.16	1372	CH deformation (cellulose and hemicellulose)
1436.80	1430–1485	C-H deformation, asymmetric in –CH_3_ and
		–CH_2_– (cellulose)
	1440	aromatic C=C stretching
	1400–1600	C=C stretch
1507.82	1514	Secondary aromatic amines
	1400–1600	C=C stretch
1560.27	1580–1590	COO- asymmetric stretching
	1400–1600	C=C stretch
	1560	COO-asymmetric stretching vibrations
1577.71	1580–1590	COO- asymmetric stretching
	1400–1600	C=C stretch
	1587	C=O
	1597	Aromatic skeletal vibration with C=O stretching vibration
1683.91	1620–1650	Aromatic and olefinic C=C vibrations, C=O amide(I), ketone and quinone groups
	1676	C=O vibration in carboxylic group of phenolic acids (p-hydroxybenzoic acid)
	1600–1700	C=C and C=O stretching
2342.48	2332	CO_2_ adsorption, Carbonyl bond group
2360.46	2332	CO_2_ adsorption, Carbonyl bond group
2917.86	2850–2950	C-H stretching of alkyl structures
	2916–2936	–CH_2_– asymmetric stretch
3420.33	3400–3410	H-bonded O-H stretching vibrations of hydroxyl groups from alcohols, phenols, and organic acids
	3428–3437	O-H stretch
3566.46–3853.46	3200–3550	O-H

**Table 3:** Isotherm models parameters for polyphenols adsorption by GPRs biochar.

Type of Isotherm	Parameters	CA	GA	OLP
Langmuir	q_max_ (mg g^−1^)	8.21	118	45.5
	K_L_ (L mg^−1^)	0.08	0.24	0.39
	R_L_ R^2^	0.02–0.71 0.9627	0.04–0.45 0.9670	0.05–0.34 0.9934
Freundlich	K_F_ (mg g^−1^) × (L g^−1^)^n^ 1/n	0.80 0.62	20.6 0.70	11.3 0.66
	R^2^	0.9380	0.9373	0.9928

## Data Availability

Not applicable.
